# The Impact of COVID-19 Incidence on Motivation to Participate in a Triathlon

**DOI:** 10.3390/ijerph19095576

**Published:** 2022-05-04

**Authors:** Joanna Poczta, Nuno Almeida, Małgorzata Paczyńska-Jędrycka, Ewa Kruszyńska

**Affiliations:** 1 Faculty of Sport Sciences, Poznan University of Physical Education, 61-871 Poznan, Poland; 2 CiTUR, ESTM, Polytechnic of Leiria, 2411-901 Leiria, Portugal; nunoalmeida@ipleiria.pt; 3 Faculty of Physical Culture and Health, The University of Szczecin, 70-453 Szczecin, Poland; malgorzata.paczynska-jedrycka@usz.edu (M.P.-J.); ewa.kruszynska@usz.edu.pl (E.K.)

**Keywords:** COVID-19, sport participation, health, physical activity, motivation

## Abstract

The COVID-19 pandemic has caused many changes that have influenced the lives of people around the world and have left their mark in the world of sports, as well. Numerous restrictions resulted in the cancellation of the organization of many sports events, and the players themselves had restricted access to training. The main goal of the study was to identify the motivation to participate in a triathlon between athletes who have undergone COVID-19, those who have never been infected and those who do not know if they have ever caught the virus and to evaluate the differences between them. The assessment of the motives for participating in a sports event was conducted according to four types of orientation: social, experience, factual and results to check what benefits for well-being and self-improvement are brought about by participation in a triathlon as a mass sports event. The desire to feel unity, to develop passion, to integrate with other people and to get away from everyday life were the most important motives for those who have never been infected. It turns out that 100% of the respondents who had a history of COVID-19 disease indicated the desire to prove themselves. The desire to maintain good physical condition and health was reported by the majority of researched people, but again, all respondents (100%) who had been infected with COVID-19 indicated these motives as the most important.

## 1. Introduction

A report published in 2017 showed that cardiovascular diseases were the main cause of illness and death in Poland. The most common causes of myocardial infarction, heart failure and stroke were the way and quality of life and atherosclerosis risk factors [1,2]. World Scientific societies have published guidelines on cardiovascular disease prevention [3]. The most important recommendations concerning the prevention of cardiovascular disease were tobacco avoidance, proper diet and regular physical activity (PA), which has multidirectional health benefits [1,2,3,4,5,6]. Physical inactivity (PI) is closely associated with many chronic non-communicable diseases and even with premature mortality [7]. Many authors like Corbin, Lindsey and Welk (2001) or Golden, Williams and Ford (2004) also indicate it as a cause for mental illness [8,9]. There is an obvious negative effect on the cardiovascular system [10], as well as the risk of development of diabetes [11], osteoporosis [12], cancer and other diseases [13]. On the other hand, a high level of PA helps control body weight [14] and improves quality of life [15]. According to the recommendations of the World Health Organization (WHO) and other governmental and public health organizations, PA of adults should last at least 30 min a day, and children should be physically active for at least one hour a day [2,7].

In 2019, the approach to medical care suddenly changed, not only in Poland but also around the world. The coronavirus (COVID-19) pandemic has exerted influence on thinking about healthcare and the approach to the health of all mankind. Therefore, the concentration of activities promoting PA has become one of the most important public health strategies. The COVID-19 outbreak has become an international public health threat [16] and has prompted governments in various countries to take swift and protective measures such as closing down cities [17], implementing and enforcing travel warnings/bans [18], extending public holidays, closing schools and postponing classes [19], as well as directing people to stay at home as a primary means of reducing their exposure to the virus. Staying at home became a basic safety measure that could limit the spread of infection. However, it has exacerbated behaviors that lead to inactivity and contribute to anxiety and depression, a sedentary lifestyle and, consequently, to a range of chronic conditions. Maintaining regular PA and an exercise routine in a safe home environment is an important strategy for healthy living during the coronavirus crisis [20]. Currently, there are studies available on the effects of COVID-19 on the mental and physical health of people around the world. The first studies were carried out in China. It was found that at the start of the COVID-19 epidemic, more than half of the respondents rated its psychological impact as moderate to severe, and around a third reported moderate to severe anxiety [21]. There was an increase in negative emotions and sensitivity to social threats, while there was a decrease in positive emotions and life satisfaction. People cared more about their family and health and less about friends and recreation [22]. The decrease in PA was related to isolation, which, in the first stage of the pandemic, made it impossible to run, cycle or walk. The organization of mass sporting events such as half marathons, marathons and triathlons was also suspended. Such restrictions on competition resulted in a decrease in the motivation for running [23]. Some studies showed that the psychological effects of the COVID-19 pandemic are visible in various social groups and the lack of motivation to undertake any activity, apart from symptoms of anxiety, depression or post-traumatic stress disorder, is one of the most common causes of its occurrence [24,25,26,27].

The dynamic development of mass sports events and the popularization of running around the world were observed long before the COVID-19 pandemic. At that time, there were also numerous research results that answered questions about the motivation to participate in them [28,29,30]. Running was an extremely popular sport and leisure activity [31], but before the outbreak of the pandemic, another new trend was also visible: mass participation in the triathlon [32]. There has been a shift in the popularity of mass participation in running events and the need to evoke strong emotions in sports participation. Runners began to seek these emotions in more difficult sports, and the triathlon, which consists of swimming, cycling and running, is a more demanding discipline [33]. People who practice in triathlons are characterized by very good health, physical condition and endurance.

The triathlon is also an endurance discipline. The competition begins with swimming in an open water area. Late in the transition zone, the players change their clothes and go to the cycling stage, then the last stage—running—begins in the next transition zone. The transition zones are the areas where riders change clothes and equipment after the swimming stage (T1) and after the cycling stage (T2). The time spent in the zones counts towards the full race time. The ability to make quick and effective changes is also an element of triathletes’ training. The first triathlon competition took place on 25 September 1974 in San Diego, with a distance of 457.2 m in swimming, 8.047 km by bike and 9.656 km in running. In 2000, the triathlon entered the Olympic Games in Sydney as an Olympic discipline; the three stages were played at distances of 1.5 km, 40 km and 10 km, respectively. The first triathlon competition in Poland took place in Poznan on Lake Kiekrz on 14 July 1984; the three stages were played at distances of 1.5 km, 50 km and 20 km. The triathlon is an individual sport; in most cases it is forbidden to help volunteers providing, for example, food on the route. Some Ultra competitions are an exception. Contrary to classic road cycling, team tactics are forbidden in the triathlon, i.e., riding in the wind tunnel created by the competitor in front, known as drafting, hence the necessary distance between players is usually required. Time penalties are charged for any breaches of the regulations. Individual professions are usually characterized by individual regulations and rules for individual stages. Unfortunately, the isolation caused by COVID-19 and the travel ban make training and participation in triathlons significantly more difficult. Bearing in mind that a triathlete’s training is quite diverse, as it must include training units focusing on at least three basic elements: swimming, cycling and running, it is much easier to find enough space just for running, which can be performed at night [34]. The coronavirus pandemic and the spread of COVID-19 have severely restricted our everyday sports lives. Closed swimming pools, gyms, fitness rooms, canceled group training sessions—all this disturbs the cycle of preparations for the season. In Poland, due to the climate, there are no conditions for swimming in open waters all year round. The vast majority of triathletes do not have a counter-current pool at home. Competitors lost the opportunity to improve their crawl technique and to build efficiency and speed in the water. Moreover, many triathletes use group activities such as CrossFit and station circuits. This form of training was out of the question for the duration of the pandemic. Therefore, another question arises about the motivation to participate in triathlons during the COVID-19 pandemic. Do athletes who have undergone COVID-19 have concerns or reluctance towards this type of PA? Does motivation change depending on exposure to the virus? What is the impact of the pandemic on the triathlon? Investigating the motivation to participate in triathlons during a pandemic turned out to be extremely interesting, but also constitutes a research niche.

## 2. Research Goal

The main objective of the study was to identify the motivation to participate in the triathlon among athletes who have undergone COVID-19, those who have never been infected and those who do not know if they have ever had the virus, and to evaluate the differences between them.

## 3. Research Project

Four main types of orientation among motives for participation in sports events distinguished by Freyer and Gross [35] were the basis of the research and the creation of the research tool: (a) social orientation, which is focused on the relationships of visitors to each other; (b) sensation-seeking orientation, which most often concerns positive experiences, in the form of, for example, relaxation, which is a kind of compensation for the hardships of everyday life; (c) factual orientation/sports discipline orientation, referring to the sports events themselves and their specificity; in this case, to the specificity of running; and (d) result orientation, which is triggered by the need to identify with success, and in the case of failure, by the need to show sympathy and solidarity.

The research was conducted from January until March 2021, using an anonymous, voluntary and confidential diagnostic survey method with the use of a standardized interview technique. All respondents (n = 1141) first declared participation in a triathlon. The sample consisted of 1141 triathletes: 721 males and 420 females. We were unable to conduct the interviews in person and meet the respondents. The limitation of the research was caused by the COVID-19 pandemic, so online survey forms were sent to the organizers of the Super League Triathlon in Poland, and with their cooperation, data collection was conducted. All respondents first declared participation in a triathlon, and we reached them with the help of the organizer of the Super League Triathlon in Poznan. They were also members of triathlon societies and sports clubs. The sample was selected in a way that would ensure the best representativeness of the obtained results. The scheme of simple random sampling without replacement was used. In determining the number of athletes, data from the organizer of the Super League Triathlon in Poland on the expected number of participants in the events organized in Poland each year was used. All questionnaires were complete. Moreover, the questionnaire was in Polish, therefore only Polish triathletes could complete it. In calculations, the formula for the sample size for a finite population was used. The assumption was made that the maximum error of estimate (e) at a 95% confidence level should not exceed 4%. The questionnaire was divided into two parts: socio-demographic variables and motives for participating in sporting events. The results, consisting of different types of motives, often exceeded 100% because, in each group of motives, participants could tick more than one answer (a maximum of three answers). Respondents were informed about the nature and goal of the survey. The study was conducted in conformity with the Declaration of Helsinki, with all participants treated according to the American Psychological Association’s ethics code. The research tool was validated during the 5th and 6th Poznan Half Marathons.

## 4. Participants

Most of the respondents (n = 1141) were between 41–50 years of age (41.36%—472), 36–40 years of age (40.6%—343) and 25–36 years of age (n = 160, 14.02%). Triathletes with higher education constituted the vast majority, 80.63% (920), and 88.95% (1015) were professionally active. Table 1 presents the socio-demographic characteristics.

Most of the women (191) were aged 36–40 (45.47%), with most of them (352) having completed higher education (83.80%), and over 80.00% (336) of the female participants were professionally active. Younger women took part in triathlons more often than men. More women than men suffered from COVID-19 infection (33.81%). Among the surveyed men (n = 721), the majority of runners were aged between 41 and 50 (51.17%—369), and then between 36 and 40 (20.94%—151). Men with higher education constituted the vast majority—78.77% (568)—while 10.67% (77) had incomplete higher education. A great percentage—94.03% (678)—were professionally active. However, twelve female respondents were reluctant to answer questions about COVID-19 infection. Therefore, the answers of 1129 respondents (721 men and 408 women) were analyzed, and it follows that more women than men became ill due to COVID-19. Only 178 male athletes (24.69%) in comparison to 33.81% of women suffered from COVID-19 infection in the research group.

## 5. Data Analysis

To verify whether respondent’s viral infection had a significant impact on their motives for participating in the event, chi-square tests were carried out, and with significant results, the Phi coefficients were calculated to determine the strength of this relationship. Statistical significance was set at *p* ≤ 0.05. Describing strength of association, it is assumed that: >0.5 is high association, 0.3 to 0.5—moderate association, 0.1 to 0.3—low association and 0 to 0.1—little if any association. To interpret Cramer’s V, the following approach is often used: V ≤ (0.1, 0.3): weak association, V ≤ (0.4, 0.5): medium association and V > 0.5: strong association. All statistical analyses were conducted using Statistica Software 10.0 (StatSoft Inc., Cracow, Poland, 2011).

## 6. Results

The main goal of the study was to identify the motivation to participate in the triathlon among athletes who have undergone COVID-19, those who have never been infected and those who do not know if they have ever had the virus, and to evaluate the differences between them. We wanted to check whether these three factors affect motivation to participate in triathlons, especially since the difficult epidemiological situation causes many barriers, such as: reaching the starting point, travel bans, the need to vaccinate or fear of infection. We were also wondering whether the fear for their own health after a history of COVID-19 might be motivating. In the first group of motives (Table 2), the most important factors for all respondents were: the desire to feel unity and integrate with other people (320 triathletes), none of the motives listed in this group (708), the desire to gain recognition in the eyes of others (151 respondents) and the sense of belonging to the subculture of runners (109).

The selections between those polled and those who had contracted COVID-19, those who had never been infected or those who did not know if they had had been infected with the virus showed a similar result. However, the desire to feel unity and integration with other people was the most important motive for the never-been-infected group, with 42.2% of all respondents stating that. To verify whether the respondents’ COVID-19 experience had a significant impact on their motives for participation in events, chi-square tests were carried out, and the Cramer’s V coefficients were calculated to determine the strength of this relationship. All relationships turned out to be significant, with a weak or moderate effect.

The next group of motives (Table 3), “within the scope of experience orientation”, shows that three motives are the most important for the respondents. The first one is the desire to experience strong emotions associated with participation in the triathlon—750 respondents (65.73%). The next one is the desire to experience a unique mood during the event—declared by 51.28% (579) of people. The third is the desire to have fun—important for 51.01% (576).

When analyzing the data in Table 3, we notice that for people who have had COVID-19, the desire to have some enjoyable leisure time is a more important motive than for those who have never been infected, but for these groups of athletes, it is more important to get away from everyday life. To verify whether the respondents’ COVID-19 experience had a significant impact on their motives for participation in the event, chi-square tests were carried out and the Cramer’s V coefficients were calculated to determine the strength of this relationship. All dependencies, apart from the desire to experience the joy and the attractiveness of the city, turned out to be significant, with a very weak or weak effect.

In the motives within the scope of factual orientation (Table 4), the desire to develop passion was important for 77.77% of all athletes (878) but the most important for those who had never been infected by COVID-19 (82.8%) and for those who did not know if they had been infected (81.5%). Moreover, half of the competitors (55%) took part in a triathlon because of the attractiveness of the sports part of the event. This motive was more important for those who had been infected by COVID-19 (61.9%).

To verify whether the respondents’ COVID-19 experience had a significant impact on their motives for participation in the event, chi-square tests were carried out and the Cramer’s V coefficients were calculated to determine the strength of this relationship. All dependencies in Group C turned out to be significant, with little effect.

The most frequently indicated motive in group D (Table 5) in terms of the result orientation for all respondents was the desire to test themselves (at the level of 87.24%—985). Then, an essential and highly rated motive was the desire to achieve the avowed goal—indicated by 77.41% (874) of the surveyed athletes.

Participation in a sports competition was more important for people who had not been infected by COVID-19 (60.7%) than for those who had (40.6%) and those who did not know if they had contracted the virus (28.3%). In this study, it turns out that 100% of respondents who had a history of COVID-19 disease indicated the desire to prove themselves. However, the high (international) rank of the sporting event did not matter in the slightest to them.

To verify whether the respondents’ COVID-19 experience had a significant impact on their motives for participation in the event, chi-square tests were carried out and the Cramer’s V coefficients were calculated to determine the strength of this relationship. In the case of failure to meet the assumption regarding the expected counts—insufficient sample size—Fisher’s exact test was used. All relationships turned out to be significant, with a very weak, weak or moderate effect.

The desire to maintain good physical condition and health (Table 6) was reported by 91.5% of the respondents, but again, all respondents (100%) who had been infected with COVID-19 indicated these motives as important.

To verify whether the respondents’ COVID-19 experience had a significant impact on motive for participation in a triathlon, chi-square tests were carried out, and the Cramer’s V coefficients were calculated to determine the strength of this relationship. The dependencies turned out to be significant, with little effect.

## 7. Discussion

Physical inactivity (PI) is a global problem. Numerous studies and reports show that every fourth adult is not active enough to achieve health benefits. This, of course, substantially increases the risk of developing non-communicable diseases [36]. Participation in universal sport has faced many restrictions since the start of the COVID-19 pandemic. Introducing isolation, enforcing travel restrictions and banning meetings and the organization of events are just a few of them. People began to fear for their health, therefore, places where sports meetings were held were avoided. Moreover, access to training sessions was also difficult.

It turns out that taking up PA during a pandemic is still essential for people who are very active. Research among triathletes shows that they want not only participation in sports events, but also to feel unity and integration with others and to get away from everyday life as well as to develop their passion.

Some people feel fear for their health and show a desire to test themselves. These are respondents who have undergone COVID-19. Therefore, the psychological consequences of a pandemic have many dimensions. However, the respondents who had not been ill in the past or did not know whether they had been infected or not had a much lighter approach and felt the desire to have some enjoyable leisure time by participating in a mass sporting event.

People behave conservatively and aloof when they feel threatened by disease. This phenomenon is closely related to the Behavioral Immune System Theory (BIS) [37]. According to this theory, there is a strong belief that people can just develop negative emotions (e.g., aversion, fear, etc.) [38,39] and negative cognitive assessments [40,41] because they want to protect themselves. In the face of the risk of disease, people tend to self-protect, that is, they increase avoidance behaviors, such as limiting contacts with people who suffer from symptoms similar to pneumonia [42]. They also enhance conformist behavior that strictly adheres to social norms [43]. According to the theory of stress [44] and the theory of perceived risk [45], crises related to public health cause many negative emotions and have a negative impact on the assessment of the surrounding situation. However, long-term isolation and strong negative emotions weaken people’s immune functions and destroy the balance of physiological mechanisms [46]. Therefore, it is important to understand the potential psychological changes caused by COVID-19.

Staying at home, while safe, can have unintended negative psychological consequences and more. Efforts to avoid human-to-human transmission of the virus also lead to a reduction in PA. A prolonged stay at home leads to an increase in sedentary behavior, which in turn leads to an increased risk and potential aggravation of chronic diseases. Official measures to restrict people’s movement in the wake of the coronavirus crisis do not necessarily mean restricting PA.

## 8. Conclusions

In our study on the motives for participating in triathlons among those who had contracted COVID-19, those who had never been infected or those who did not know if they had contracted the virus, we have obtained different results. The desire to feel unity and integration with other people was the most important motive for those who had never been infected. Moreover, getting away from everyday life was more important for them, too. The desire to develop passion was important for all athletes, but the most important for those who had never been infected by COVID-19 and for those who didn’t know if they had been infected. For people who underwent COVID-19, the desire to have some enjoyable leisure time was a more important motive than for those who had never been infected. Participation in a sports competition was more important for people who had not been infected by COVID-19 than for those who had and those who didn’t know. In this study, it turns out that 100% of respondents who had a history of COVID-19 disease indicated the desire to prove themselves. However, the high (international) rank of a sporting event did not matter in the slightest to them. The desire to maintain good physical condition and health was reported by the majority of the respondents but again, all respondents (100%) who had been infected with COVID-19 indicated these motives as important.

## Figures and Tables

**Table 1:** Socio-demographic characteristics of respondents.

Socio-Demographic Characteristics	Male	%	Female	%	All	%
	N = 721		N = 420		N = 1141	100
Age						
<18	13	1.80	25	5.95	38	3.33
19–24	13	1.80	16	3.80	29	2.54
25–35	101	14.00	58	13.80	160	14.02
36–40	151	20.94	191	45.47	343	30.06
41–50	369	51.17	103	24.52	472	41.36
51–70	73	10.12	26	6.19	99	8.67
Education Level						
Primary education	13	1.80	12	2.85	25	2.19
Secondary education	63	8.73	27	6.42	90	7.88
Incomplete higher education	77	10.67	29	6.90	106	9.29
Completed higher education	568	78.77	352	83.80	920	80.63
Employment Status						
School pupil (<18 years)	26	3.39	12	2.85	38	3.33
University student	0	0.00	29	6.90	29	2.54
Professionally active	678	94.03	336	80.00	1015	88.95
Unemployed	0	0.00	30	7.14	30	2.62
Pensioner	16	2.21	13	3.09	29	2.54
Suffered COVID-19 infection	721		408		N = 1129	
Yes	178	24.69	142	33.81	320	28.04
No	324	44.93	200	47.62	512	45.93
I don’t know	219	30.38	78	18.57	297	26.03

**Table 2:** Motives within the scope of social orientation versus COVID-19 infection.

Group A	They Were Infected (n = 320)		They Do Not Know (n = 297)		They Have Not Been Infected (n = 512)		All (n = 1129)	*p*	Cramer’s V
	n	%	n	%	n	%			
Desire to feel unity and integration with other people	50	15.6	54	18.2	216	42.2	320	<0.001	0.28
Desire to feel equality during the race	22	6.9	0.0	0.0	68	13.3	90	<0.001	0.20
Prevailing fashion—participation in sports events is currently fashionable	32	10.0	0.0	0.0	0.0	0.0	32	<0.001	0.27
Desire to gain recognition in the eyes of others	32	10.0	26	8.7	93	18.2	151	<0.001	0.13
Belonging to the subculture of runners	32	10.0	0	0.0	77	15.0	109	<0.001	0.21
None of the motives listed in this group	238	74.4	243	81.8	227	44.3	708	<0.001	0.35

**Table 3:** Motives within the scope of experience orientation versus COVID-19 infection.

Group B	They Have Been Infected (n = 320)		They Do Not Know (n = 297)		They Have Not Been Infected (n = 512)		All (n = 1129)	*p*	Cramer’s V
	n	%	n	%	n	%			
Desire to experience strong emotions associated with participation	247	77.2	146	49.2	357	69.7	750	<0.001	0.23
Desire to experience the unique amosphere during the event	163	50.9	182	61.3	234	45.7	579	<0.001	0.13
Desire to have good fun	199	62.2	112	37.7	265	51.8	576	<0.001	0.18
Desire to have enjoyable leisure time/entertainment	88	27.5	40	13.5	85	16.6	213	<0.001	0.14
Desire to express happiness, e.g., resulting from winning/success	28	8.8	42	14.1	50	9.8	120	0.066	-
Desire to get away from everyday life	62	19.4	86	29.0	120	23.4	268	0.020	0.08
Being allured by the attractiveness of the city in which the event takes place	19	5.9	26	8.8	30	5.9	75	0.235	-
None of the motives listed in this group	13	4.1	41	13.8	44	8.6	98	<0.001	0.13

**Table 4:** Motives within the scope of factual orientation versus COVID-19 infection.

Group C	They Have Been Infected (n = 320)		They Do Not Know (n = 297)		They Have Not Been Infected (n = 512)		All (n = 1129)	*p*	Cramer’s V
	n	%	n	%	n	%			
Desire to develop passion	212	66.3	242	81.5	424	82.8	878	<0.001	0.18
Being drawn by the attractiveness of the sports part of the triathlon	198	61.9	132	44.4	291	56.8	621	<0.001	0.13
Being drawn by the attractiveness of the extensive program of the accompanying events	0	0.0	0	0.0	40	7.8	40	<0.001	0.21
None of the motives listed in this group	20	6.3	41	13.8	34	6.6	95	<0.001	0.12

**Table 5:** Motives within the scope of result orientation versus COVID-19 infection.

Group D	They Have Been Infected (n = 320)		They Do Not Know (n = 297)		They Have Not Been Infected (n = 512)		All (n = 1129)	*p*	Cramer’s V
	n	%	n	%	n	%			
Desire to test oneself	320	100.0	202	68.0	463	90.4	985	<0.001	0.37
Desire to achieve the avowed goal	227	70.9	212	71.4	435	85.0	874	<0.001	0.16
Desire to participate in sports competition	130	40.6	84	28.3	311	60.7	525	<0.001	0.28
Desire to win	22	6.9	30	10.1	65	12.7	117	0.027	0.08
High (international) rank of this sporting event	0	0.0	31	10.4	16	3.1	47	<0.001	0.20
None of the motives listed in this group	0	0.0	13	4.4	0	0.0	13	<0.001	0.18

**Table 6:** Other motives versus COVID-19 infection.

Group E	They Have Been Infected (n = 320)		They Do Not Know (n = 297)		They Have Not Been Infected (n = 512)		All (n = 1129)	*p*	Cramer’s V
	n	%	n	%	n	%			
The desire to maintain good physical condition and health	320	100.0	255	85.9	458	89.5	1033	<0.001	0.20

## Data Availability

The data presented in this study are available on request from the corresponding author. The data are not publicly available.
